# Splenic infarction after Epstein–Barr virus infection in a patient with hereditary spherocytosis: a case report and literature review

**DOI:** 10.1186/s12893-022-01580-5

**Published:** 2022-04-09

**Authors:** Zhongwu Ma, Zhejin Wang, Xiaodan Zhang, Haibo Yu

**Affiliations:** Department of Hepatobiliary Surgery, Wenzhou Central Hospital, The Dingli Clinical Institute of Wenzhou Medical University, Wenzhou, 325000 Zhejiang People’s Republic of China

**Keywords:** Hereditary spherocytosis, Splenic infarction, EBV, Laparoscopic splenectomy

## Abstract

**Background:**

Hereditary spherocytosis (HS) complicated by splenic infarction is very rare, and it is even rarer to develop splenic infarction after infectious mononucleosis (IM) as a result of Epstein–Barr virus (EBV) infection. Therefore, misdiagnosis or missed diagnosis is prone to occur.

**Case presentation:**

A 19-year-old Chinese female previously diagnosed with HS was admitted to our institution with persistent high fever and icterus. On admission, the physical examination showed anemia, jaundice, marked splenomegaly, obvious tenderness in the left upper abdomen (LUA). Peripheral blood film shows that spherical red blood cells accounted for about 6%, and Immunoglobulin M (IgM) antibodies specific to Epstein–Barr virus (EBV) viral capsid antigen were detected. An abdominal CT scan revealed a splenic infarction. The patient was diagnosed with HS with splenic infarction following EBV infection and underwent an emergency laparoscopic splenectomy (LS). Pathological analysis showed a splenic infarction with red pulp expansion, white pulp atrophy and a splenic sinus filled with red blood cells. After two months of follow-up visits, the patient showed no signs of relapse.

**Conclusions:**

HS complicated by splenic infarction is very rare and mostly occurs in men under 20 years of age and is often accompanied by other diseases, such as sickle cell traits (SCT) or IM. Although symptomatic management may be sufficient, emergency laparoscopic splenectomy may be safe and effective when conservative treatment is ineffective.

## Background

Hereditary spherocytosis (HS) is a heterogeneous hereditary hemolytic anemia resulting from defects in genes encoding the red cell membrane skeleton [1]. The main clinical characteristics of HS include anemia, intermittent jaundice, splenomegaly and cholelithiasis. However, there is a notable heterogeneity in clinical characteristics with hemolytic anemia varying from mild to fulminant [2]. HS is characterized by the presence of spherocytes on the peripheral blood smear, which are formed by defects of erythrocyte membrane protein, such as spectrin and Ankyrin [1, 3]. The main cause of hemolysis in HS is the selective isolation and destruction of spherocytes in the spleen. Familial studies indicate that HS often arises due to autosomal dominant inheritance, accounting for about 75%.

Infectious mononucleosis (IM) is an acute self-limiting infectious disease caused by the Epstein–Barr virus (EBV) [4]. According to epidemiological surveys, EBV is estimated to affect more than 90% of the world's population [5]. Its main clinical features are fever, pharyngitis, lymphadenopathy, a significant increase in peripheral blood lymphocytes and abnormal lymphocytes, a positive heterophilic agglutination test, and the appearance of anti-EBV antibodies in the body after infection.

To our knowledge, the occurrence of splenic infarction in HS patients following EBV infection is quite rare. To this date, there is no literature available on cases that underwent emergency laparoscopic splenectomy(LS) as management. Herein, we report the case of an HS patient who developed a splenic infarction following an EBV infection, that was managed using emergency LS. Furthermore, a systematic review of this case was performed to offer more clinical insight and information.

## Case presentation

**Fig. 1:**
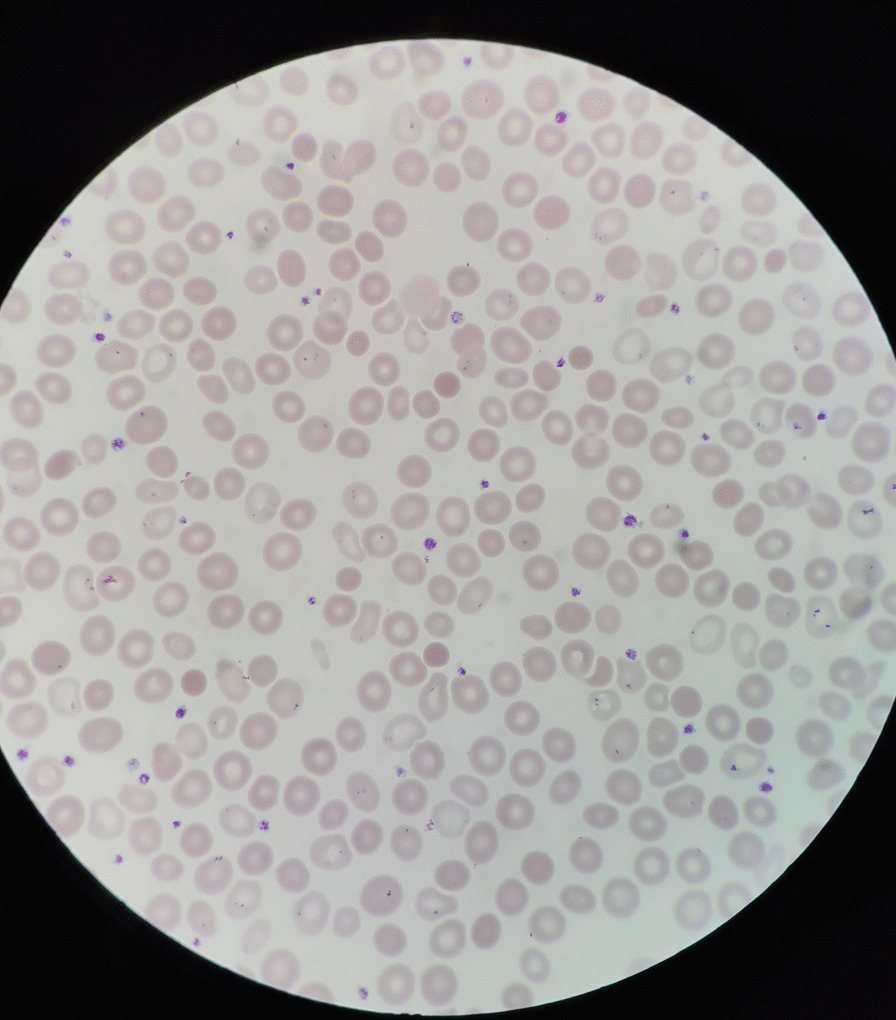
Peripheral blood film showed that spherical red blood cells accounted for about 6%

**Fig. 2:**
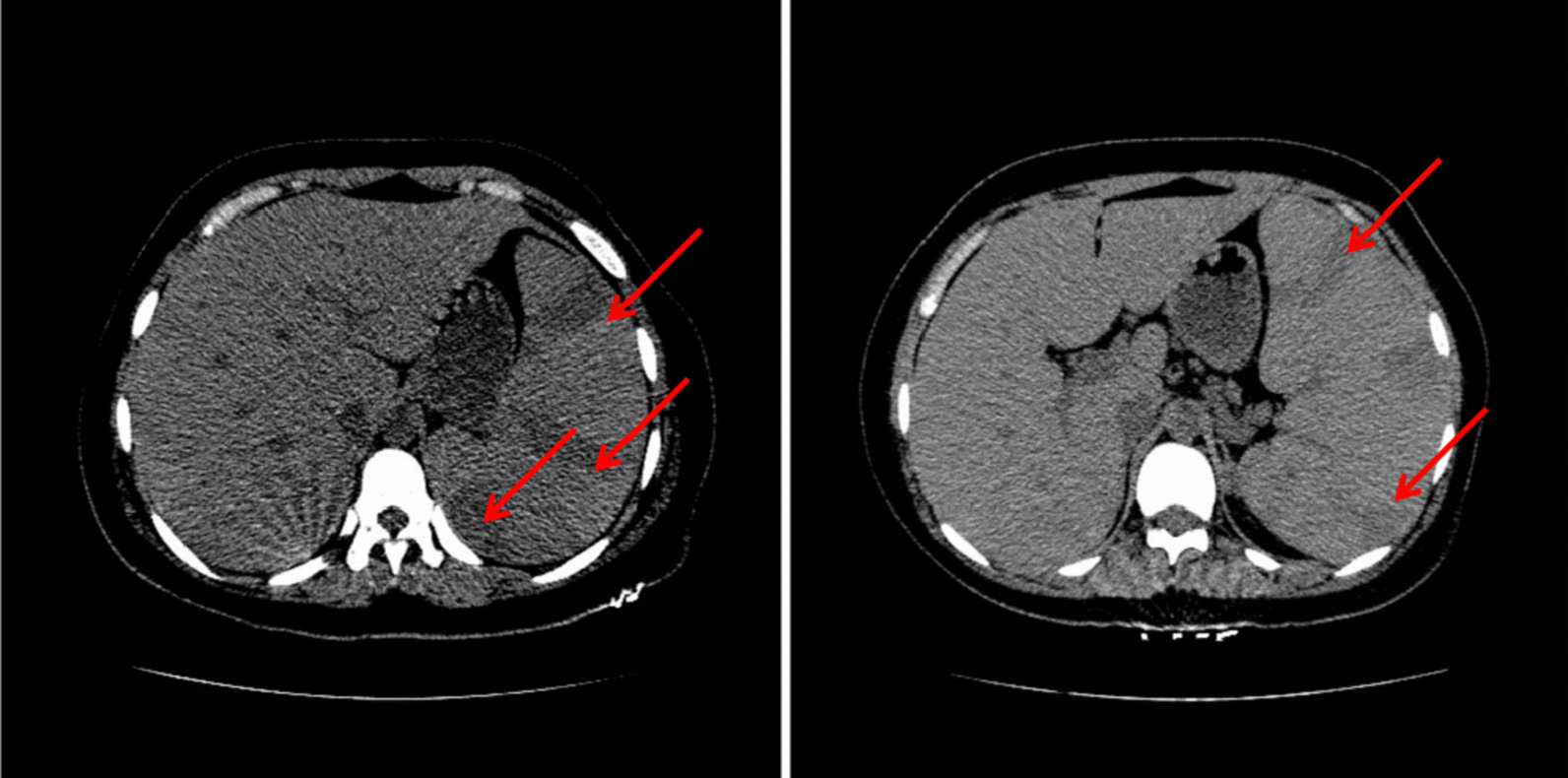
The CT at abdomen showing that the spleen was enlarged with multiple wedge-shaped low-density foci, suggesting the presence of splenic infarction.The arrows point to the splenic infarction

**Fig. 3:**
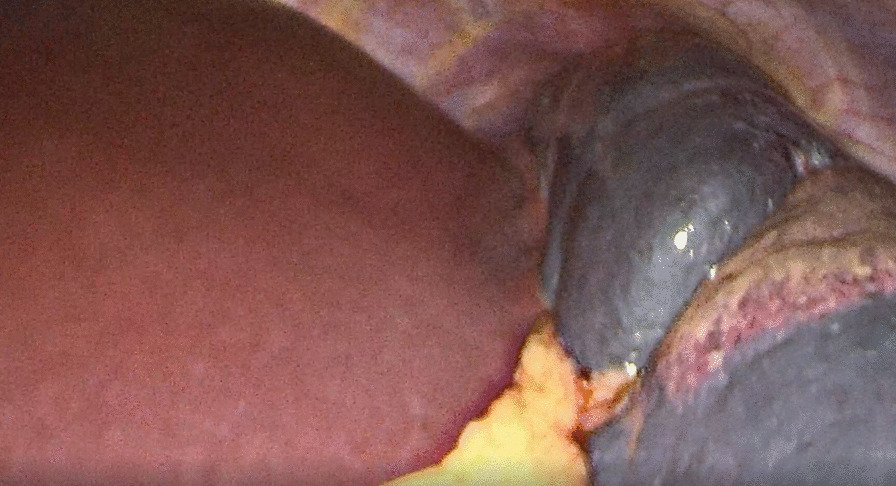
The spleen was obviously enlarged and presented with multiple large-area splenic infarcts

**Fig. 4:**
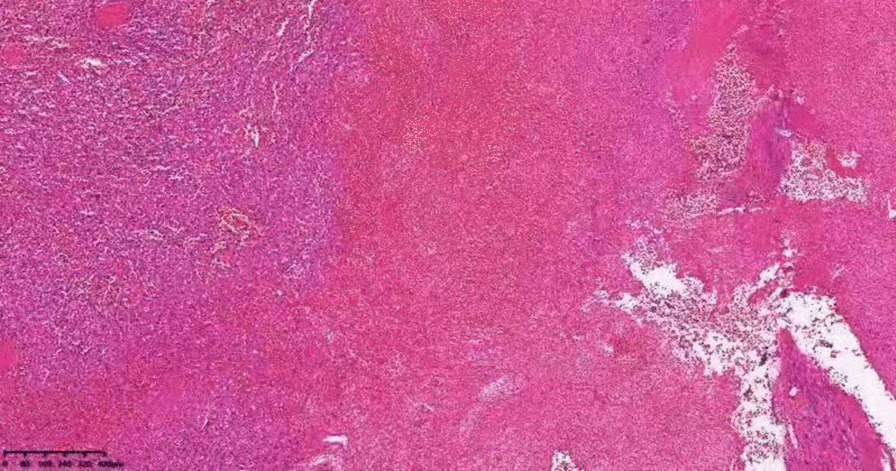
Pathology review demonstrated splenic infarction with red pulp expansion, white pulp atrophy, splenic sinus filled with red blood cells

A 19-year-old Chinese female was admitted to our institution with persistent high fever and icterus. Six months ago, she was diagnosed with HS due to icterus. HS had been diagnosed in her mother, and her symptoms, such as anemia after splenectomy, were significantly improved. One week ago, due to dizziness and fatigue, she was admitted to a local hospital for low hemoglobin (47 g/L). Red blood cell transfusion improved her anemia, with hemoglobin level rising to 62 g/L. However, symptoms of dizziness and fatigue did not alleviate. Two days ago, the patient developed fever and icterus and was admitted to our Hematology Department. On admission, physical examination findings were as follows: anemia, jaundice, marked splenomegaly, obvious tenderness in the left upper abdomen(LUA), and absence of lymphadenopathy.Laboratory tests revealed a WBC count of 3.9 × 109/L (lymphocytes 50.5%), an RBC count of 2.17 × 1012/L, a Hb concentration of 52 g/L, an HCT of 16.0%, an Rtc count of 5.8%, an atypical lymphocytes count of 7%, and a PLT count of 164 × 10^9^/L. Peripheral blood film showed that spherical red blood cells accounted for about 6% (Fig. 1). Blood biochemical analysis was as follows: TBIL 5.3 mg/dL; IBIL 4.1 mg/dL; ALT 66 U/L; AST 65 U/L; LDH 958 U/L; CPK 55.9 U/L; ferritin 1344 ng/mL; CRP 145.9 mg/L; PCT 0.46 μg/L. Coagulation tests: D-dimer 717 ug/L; PT 12 s. The results of the Coombs tests were negative. Ham and sugar-water tests were faintly positive. The result of G6PD was normal. The osmotic fragility test showed that the osmotic fragility of erythrocytes increased overall. Autoimmune workup was negative. Paroxysmal nocturnal hemoglobinuria screened by flow cytometry was negative. Serologic test results revealed a positive EBV infection (EBV viral capsid antigen (EBV-VCA) IgM-positive, EBV-VCA IgG-positive, EBV nuclear antigen (EBNA) Ig G-positive, and EBV early antigen (EA) IgM-positive). Due to obvious tenderness in the LUA, a CT scan of the abdomen was performed, and it was found that the spleen was enlarged with multiple wedge-shaped low-density foci, suggesting the presence of splenic infarction (Fig. 2). Because of the poor effect of symptomatic and supportive treatment, emergency LS was performed. Intraoperatively, her spleen was obviously enlarged and presented with multiple large-area splenic infarcts (Fig. 3). Pustular lesions were also observed on the surface of the spleen. Pathology review demonstrated splenic infarction with red pulp expansion, white pulp atrophy, splenic sinus filled with red blood cells (Fig. 4). Unfortunately, the pathological evaluation of EBV in the resected spleen was not performed. Her operative and postoperative courses were uneventful. The patient recovered quickly after the operation and was discharged accordingly. BRT was made every 2 weeks after surgery. After two months of follow-up visits, the patient showed no relapse.

## Discussion and conclusions

Splenic infarctions are seldom observed in HS. We searched for studies using PubMed Medline with a search strategy combining search terms and Medical Subject Headings (MeSH). The search strategy is as follows: (("Spherocytosis, Hereditary"[Mesh]) OR (Hereditary Spherocytoses[Title/Abstract])) AND (("Splenic Infarction"[Mesh]) OR ((((Splenic Infarction[Title/Abstract]) OR (Infarct of the Spleen[Title/Abstract])) OR (Splenic Infarct*[Title/Abstract])) OR (splenic sequestration[Title/Abstract]))). A total of 19 articles were obtained, 7 of which were excluded. A total of 13 cases with splenic infarction/sequestration associated with HS were selected, as shown in Table 1. Our research showed that HS combination splenic infarction was more common in male patients younger than 20 years old. Among these 13 patients, only 2 were patients older than 20 years [6, 7], and only 2 were female [8, 9]. The coexistence of HS and sickle cell traits(SCT) is unusual, but splenic infarction is far more likely to occur in such patients [7, 8, 10–14]. The proposed mechanism is that the concentration of hemoglobin S in the spherocytes is higher; sickling might occur and cause decreased red cell deformability in the acidotic and hypoxic environment. As a result, a large number of cells are selectively retained in the sinusoids of the spleen and eventually induce infarction [7, 13, 14]. Thus, high altitude or airplane flights may induce splenic infarction in these patients [8, 11, 12]. Splenic infarction after EBV infection in HS patients is very rare, and currently, only 4 cases have been reported [9, 15–17]. One reported case was that of a 13-year-old boy with coexisting protein C deficiency in addition to IM[15]. In these cases, the mechanism of splenic infarction was speculated to be due to insufficient blood and oxygen supply to the rapidly expanding spleen. Spherocytes are easily trapped in the splenic cord and removed by macrophages. During acute IM, proliferation and expansion of EBV-infected B-cells and activation of T-cells lead to the production of plenty of cytokines, resulting in rapid spleen enlargement [17]. Furthermore, EBV infection aggravates the hemolytic reaction, and the spleen rapidly enlarges to compensate, causing an insufficient oxygen supply to the parenchyma. The precise mechanism of splenic infarction remains elusive, and further case reports and studies are warranted.

**Table 1:** Summary of hereditary spherocytosis patients with splenic infarction: a literature review

Year of publication	Country	Age/sex	Underlying disease	Fever	Abdominal pain	Spleno- megaly	Splenic rupture	Splenectomy	Urgent/ selective
2007 [17]	Japan	10–20/M	IM	YES	LUA pain	YES	NO	YES	SEL
2015 [16]	United Kingdom	10–20/M	IM	YES	Left chest pain	YES	NO	NO	
2020 [9]	USA	10–20/F	IM	YES	LUA pain	YES	NO	NO	
2011 [10]	United Kingdom	10–20/M	SCT	NO	LUA pain	YES	NO	NO	
2007 [8]	USA	10–20/F	SCT	NO	LUA pain	YES	NO	YES	URG
2003 [7]	USA	40–50/M	SCT	NO	LUA pain	YES	NO	NO	
1997 [12]	France	10–20/M	SCT	YES	Left chest pain	N/A	NO	NO	
1992 [14]	USA	0–10/M	SCT	NO	LUA pain	YES	NO	YES	SEL
1992 [14]	USA	10–20/M	SCT	NO	LUA pain	YES	NO	YES	SEL
1990 [13]	USA	10–20/M	SCT	YES	LUA pain	YES	NO	YES	SEL
2008 [15]	Germany	10–20/M	IM and PCD	YES	LUA pain	YES	NO	NO	
2016 [6]	China	20–30/M	INS	YES	Left chest pain	YES	NO	YES	URG
2017 [18]	Swit	10–20/M	NO	NO	LUA pain	YES	NO	YES	URG
The present patient	China	10–20/F	IM	YES	LUA pain	YES	NO	YES	URG

*IM* Infectious mononucleosis, *SCT* Sickle cell traits, *PCD* Protein C deficiency, *INS* invasive nontyphoidal salmonellosis, *LUA* Left upper abdomen

According to the 2011 Guidelines on HS [3], the diagnosis of HS in our patient was definitive since our patient has a family history of HS, typical clinical features and laboratory investigations. Isolated splenic infarction can occur in the case of hematological malignancies, embolic diseases, splenic torsion, aortic dissection, vasculitis or vasospasm secondary to vasopressor therapy [15]. At present, there is only one reported case of a HS patient who underwent partial splenectomy due to a rare complication of secondary splenomegaly, traumatic splenic torsion, leading to splenic infarction [18]. In addition, some systemic infections are known to cause splenic infarction [6, 19–21]. Reviewing previous literature about HS with concomitant splenic infarction, we found that some were accompanied by underlying prethrombotic disease SCT [7, 8, 10–14], while others occurred following IM [9, 15–17]. When splenic infarction occurs, the common clinical manifestation is left upper quadrant abdominal pain, and occasionally an atypical left chest pain may occur. When splenic infarction occurs in patients with coexisting HS and IM, in addition to the mentioned symptoms, there are often early manifestations of high fever, but no such manifestation was present in patients with coexisting HS and SCT. This is a point of identification. Our patient developed a high fever 5 days after her blood transfusion, followed by severe pain in the LUA, considering the occurrence of splenic infarction after EBV infection. Related laboratory examinations and abdominal CT scans confirmed our diagnosis. For this condition, most patients can be effectively relieved through conservative symptomatic and supportive treatment, and splenectomy may not be a necessary treatment [22].Our patient has undergone conservative treatment, including antibiotics,but the symptoms of high fever and abdominal pain have not improved. And there was a persistent increase in white blood cells, the highest white blood cell count reaches 19 × 109/L. After careful assessment of the risks and benefits, the patient underwent emergency laparoscopic splenectomy.We summarized previous reports and found that about 7 out of 13 HS patients with splenic infarction ultimately underwent splenectomy. Splenectomy is considered to be the most reliable and effective method for the treatment of HS. Although it cannot change the morphology of erythrocytes, it greatly reduces the destruction of erythrocytes and extends their lifespan, and symptoms such as anemia, jaundice, and reticulocyte levels are often significantly improved [1]. Previous literature reported on selective re-admission to the hospital for splenectomy due to concerns about the recurrence of splenic infarction [17]. Similarly, after carefully evaluating the risks and benefits, our patient underwent emergency LS. To our knowledge, there is no case report of emergency LS for HS complicated with splenic infarction. The operation was successful, and the patient recovered well. Hence, we believe that emergency laparoscopic splenectomy might be an effective and safe treatment. Nevertheless, further case reports and clinical research are warranted to substantiate our results.

In conclusion, we report a rare complication of splenic infarction in an HS patient following EBV infection. HS complicated by splenic infarction is very rare, mostly occurring in men under 20 years of age, and is more often than not accompanied by other diseases, such as SCT or IM. Symptomatic management may be sufficient; however, emergency laparoscopic splenectomy may be a safe and effective management strategy when conservative treatment is not effective.

## Data Availability

All data produced and obtained is available within the manuscript.

## Abbreviations

HS: Hereditary spherocytosis
IM: Infectious mononucleosis;
EBV: Epstein–Barr virus
LUA: Left upper abdomen
IgM: Immunoglobulin M
CT: Computed tomography
LS: Laparoscopic splenectomy
SCT: Sickle cell traits
WBC: White blood cells
RBC: Red blood cells
Hb: Hemoglobin
HCT: Hematocrit
Rct: Reticulocyte
PLT: Platelet
TBIL: Total bilirubin
IBIL: Indirect bilirubin
ALT: Alanine aminotransferase
AST: Aspartate aminotransferase
LDH: Lactate dehydrogenase
CPK: Creatine phosphokinase
CRP: C-reactive protein
PCT: Procalcitonin
G6PD: Glucose-6-phosphate dehydrogenase
BRT: Blood routine
